# UBE2M promotes cell proliferation via the β-catenin/cyclin D1 signaling in hepatocellular carcinoma

**DOI:** 10.18632/aging.102749

**Published:** 2020-02-03

**Authors:** Guang-Cong Zhang, Xiang-Nan Yu, Jia-Lei Sun, Ju Xiong, Yi-Jun Yang, Xue-Mei Jiang, Ji-Min Zhu

**Affiliations:** 1 Department of Gastroenterology, Central South University Xiangya School of Medicine Affiliated Haikou Hospital, Haikou 570100, China; 2 Department of Gastroenterology and Hepatology, Zhongshan Hospital, Fudan University, Shanghai 200030, China; 3 Shanghai Institute of Liver Diseases, Zhongshan Hospital, Fudan University, Shanghai 200030, China; 4 Department of Hepatobiliary Surgery, Hainan General Hospital, Haikou 570100, China; 5 Department of Hepatobiliary Surgery, Central South University Xiangya School of Medicine Affiliated Haikou Hospital, Haikou 570100, China; 6 Department of Gastroenterology, Hainan General Hospital, Haikou 570100, China

**Keywords:** hepatocellular carcinoma, ubiquitin-conjugating enzyme E2M, prognosis, β-catenin, proliferation

## Abstract

Upregulated ubiquitin-conjugating enzyme E2M (UBE2M) is associated with poor prognosis in malignancies; However, the phenotype and mechanism of action of UBE2M in hepatocellular carcinoma (HCC) remain elusive. Here, we report that UBE2M is overexpressed and correlated with poor prognosis in HCC patients. The UBE2M level is an independent prognostic factor for HCC patients. UBE2M knockdown inhibits HCC cell proliferation, migration, and invasion, whereas its overexpression has an opposite effect. Mechanistically, upregulated UBE2M exerts oncogenic effects by translocation of accumulated β-catenin from the cytoplasm to the nucleus, thus activating downstream β-catenin/cyclin D1 signaling. In summary, our study demonstrates a notable role of UBE2M in promoting the growth of HCC, providing a novel strategy for HCC prevention and treatment.

## INTRODUCTION

Hepatocellular carcinoma (HCC) represents one of the most common malignancies and the third leading cause of cancer-related deaths worldwide [1]. Although early diagnostic modalities and treatment utilization have improved clinical outcomes, the long-term prognosis of HCC patients remains unsatisfactory due to recurrence and distant metastasis [2, 3]. A deeper understanding of the underlying molecular mechanisms regarding carcinogenesis and progression of HCC is thus urgently demanded.

In canonical Wnt/β-catenin signaling, aberrant β-catenin expression leads to a variety of diseases, including cancers [4, 5]. Under unstimulated conditions, intracellular β-catenin is continuously degraded by a complex containing glycogen synthase kinase 3β, scaffolding protein Axin, and adenomatous polyposis coli [6, 7]. However, under stimulated conditions, accumulated β-catenin translocates from the cytoplasm into the nucleus where it acts as a co-activator of the T-cell factor/lymphoid enhancer factor family transcription factors, resulting in transcription of multiple downstream genes involved in cell cycle progression, like cyclin D1 and c-Myc [8, 9]. Since emerging evidence suggests that ubiquitin-conjugating enzymes (E2) play a critical role in both tumorigenesis and tumor progression via regulating β-catenin expression [10–13], it has raised the question whether other members of the E2 family similarly affected tumorigenesis.

E2 family is an essential component of ubiquitination cascade, which interacts with ubiquitin-activating enzymes (E1) and ubiquitin-protein ligases (E3) for coordinately recognizing and degrading specific target proteins [14, 15]. Ubiquitin-conjugating enzyme E2M (UBE2M), an essential member of the E2 family, is located in chromosome 19q13.43. Increasing evidence shows that UBE2M expression is upregulated in many malignancies, including osteosarcoma [16], urothelial carcinoma [17], and intrahepatic cholangiocarcinoma [18], suggesting that UBE2M might be associated with onset and progression of human cancers. However, the biological function and underlying mechanism of UBE2M in HCC remain undetermined.

In this context, the present study is designed to explore the significance of UBE2M in HCC. Here we showed that UBE2M was upregulated and correlated with poor prognosis in HCC patients. Furthermore, we validated that UBE2M promoted HCC proliferation by stabilizing β-catenin and upregulating cyclin D1 expression *in vitro* and *in vivo*. Our findings provide a rationale for the clinical application of UBE2M-based strategy for HCC prevention and treatment.

## RESULTS

### UBE2M enriches in HCC tissues and cell lines

**Figure 1:**
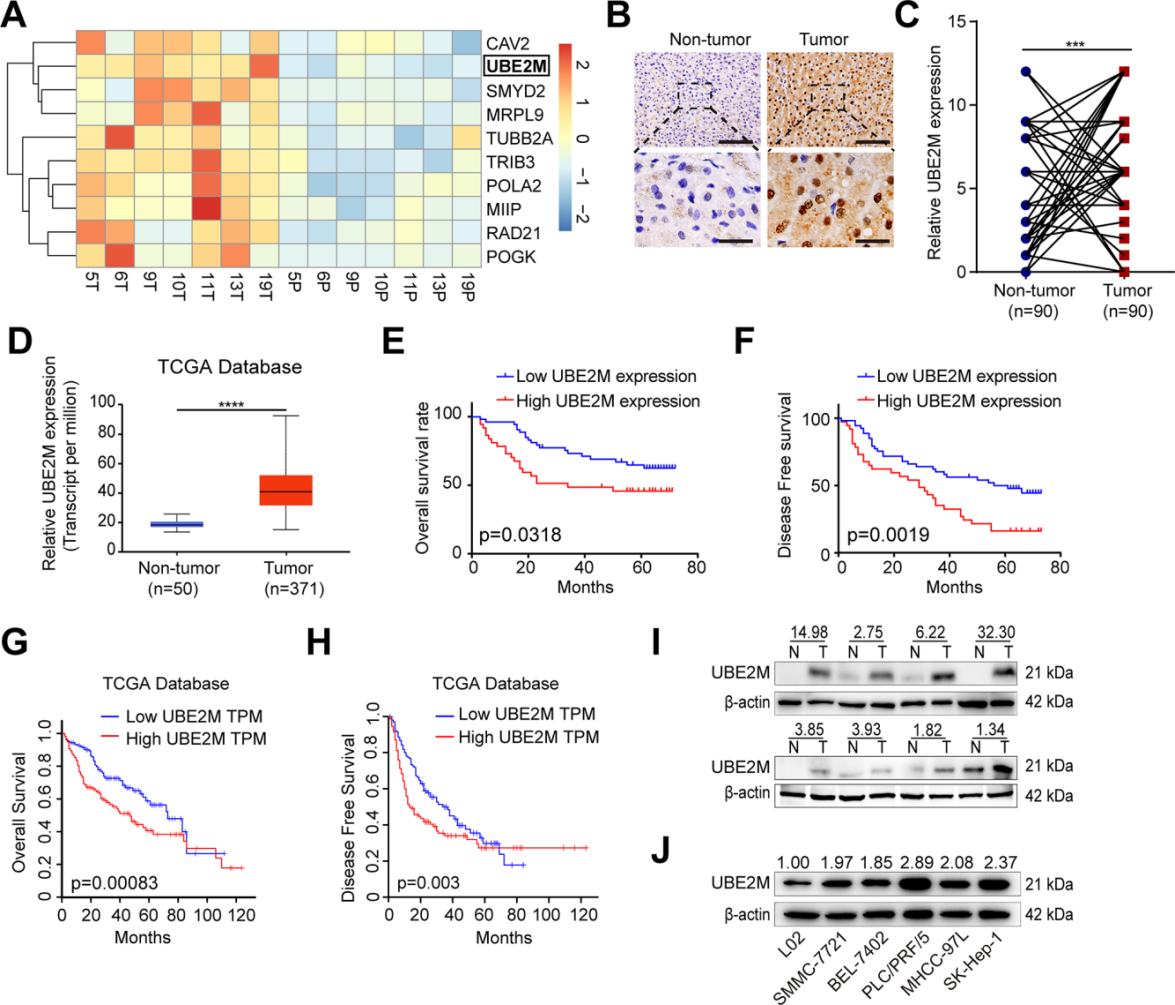
**Overexpressed UBE2M in HCC tissues and cell lines.** (**A**) Heatmap showing differential expression of genes in 7 paired HCC tissues using a mRNA microarray. (**B**) Representative images showing UBE2M expression in HCC and paired non-tumor tissues (Scale bar, 400 μm in the upper panel, and 100 μm in the lower panel). (**C**) UBE2M expression in HCC and paired non-tumor tissues using a pair-wise comparison analysis (***P < 0.001). (**D**) UBE2M expression in 371 HCC and 50 non-tumor tissues obtained from The Cancer Genome Atlas (TCGA) database (****P < 0.0001, Student’s *t*-test). Adapted from UALCAN: http://ualcan.path.uab.edu/index.html. (**E**) A Kaplan-Meier plot showing the overall survival rate of HCC patients with low- and high-UBE2M expression in our HCC cohort (*P* = 0.0318, log-rank test). (**F**) A Kaplan-Meier plot showing the disease-free survival of HCC patients with low- and high-UBE2M expression in our HCC cohort (*P* = 0.0019, log-rank test). (**G**) A Kaplan-Meier plot showing the overall survival rate of HCC patients with low- and high-UBE2M expression in TCGA database (*P* = 0.00083, log-rank test; TPM, transcript per million). Adapted from GEPIA: http://gepia.cancer-pku.cn/index.html. (**H**) A Kaplan-Meier plot showing the disease-free survival of HCC patients with low- and high-UBE2M expression in TCGA database (*P* = 0.003, log-rank test). Adapted from GEPIA: http://gepia.cancer-pku.cn/index.html. (**I**) UBE2M protein expression in 8 pairs of HCC tissues by Western blotting (N, non-tumor; T, tumor). (**J**) UBE2M protein expression in five HCC cell lines and normal hepatocytes L02 by Western blotting.

To identify the genes that highly expressed in HCC, we previously performed an Agilent Human mRNA microarray in 7 pairs of HCC specimens (GSE101728). The results showed that UBE2M was significantly overexpressed in tumor tissues as compared to matched non-tumor tissues (Figure 1A, fold change = 2.33; *P* < 0.001). Next, IHC staining on 90 pairs of HCC tissues was performed to verify UBE2M expression. We found that the UBE2M expression in HCC tissues was significantly higher than that in matched tumor-free tissues using a pair-wise comparison analysis (7.28 ± 3.66 vs. 4.02 ± 3.56; Figure 1B, 1C and Supplementary Figure 1; *P* < 0.001), which is consistent with the data in The Cancer Genome Atlas (TCGA; Figure 1D, *P* < 0.0001). Further analysis revealed that sixty-two patients have upregulated UBE2M expression in HCC compared to the non-tumor tissues. Kaplan-Meier survival curves showed that high UBE2M expression was correlated with poor overall survival (OS; Figure 1E, *P* = 0.032) and disease-free survival (DFS; Figure 1F, *P* = 0.002). Consistent with our results, analysis with TCGA dataset also demonstrated that upregulated UBE2M was correlated with a poor OS (Figure 1G, *P* = 0.00083) and DFS (Figure 1H, *P* = 0.003).

Subsequently, we examined the UBE2M expression in eight pairs of HCC specimens. The result demonstrated that UBE2M was also markedly elevated in HCC tissues compared with matched tumor-free tissues (Figure 1I). We also examined UBE2M expression in five HCC cell lines and human hepatocyte L02. The result revealed that compared with human hepatocyte L02, five HCC cell lines had significantly higher UBE2M levels (Figure 1J). These results indicate that UBE2M may act as an oncogene in HCC.

### Upregulated UBE2M correlates with clinicopathological features of HCC

To further investigate whether UBE2M overexpression is involved in HCC progression, the correlation between UBE2M and clinicopathological parameters was examined. As shown in Table 1, a high expression of UBE2M was positively correlated with cirrhosis (χ^2^ = 4.396, *P* = 0.036), tumor size (χ^2^ = 4.897, *P* = 0.027) and tumor number (χ^2^ = 5.614, *P* = 0.027). However, no correlation was observed between UBE2M expression and other clinicopathological features, including gender, age, HBsAg, serum α-fetoprotein (AFP), tumor differentiation, and TNM stage.

**Table 1:** Association of UBE2M level with clinicopathological parameters of HCC patients.

**Parameters**	**UBE2M expression**		***χ*2**	***P* value**
	**High (n=37)**	**Low (n=53)**		
Gender			0.161	0.689
Male	28	42		
Female	9	11		
Age (year)			0.357	0.551
≤55	20	32		
>55	17	21		
HBsAg			0.01	0.921
Negative	8	11		
Positive	29	42		
Cirrhosis			4.396	**0.036**
Negative	6	16		
Positive	31	27		
AFP (ng/mL)			1.469	0.226
≤20	15	15		
>20	22	38		
Tumor size (cm)			4.897	**0.027**
≤5	15	34		
>5	22	19		
Tumor number			5.614	**0.018**
Solitary	19	40		
Multiple	18	13		
Tumor differentiation			0.926	0.336
I–II	20	34		
III–IV	17	19		
TNM stage			0.293	0.588
I–II	31	42		
III–IV	6	11		

Next, the cox-regression analysis was used to determine whether UBE2M serves as a risk factor. For OS, univariate analysis revealed that HCC patients with high UBE2M expression were associated with a significantly increased risk of death compared to those with low UBE2M expression (*P* = 0.043). Multivariate analysis showed that upregulated UBE2M expression could be a significant factor for predicting poor survival (*P* < 0.01) when UBE2M expression level, TNM stage, AFP, tumor size, and tumor differentiation were included based on univariate analysis (Table 2). As for DFS, univariate analysis revealed that a high UBE2M level was associated with an increased risk of recurrence in HCC patients compared to those with low UBE2M expression (*P* = 0.007). Multivariate analysis showed that upregulated UBE2M expression could be a significant factor for predicting poor DFS (*P* = 0.008) when UBE2M expression level, TNM stage, and tumor size were included based on univariate analysis (Table 3). These results suggest that UBE2M expression is independently correlated with poor prognosis of HCC and may play an important role in HCC by promoting cell proliferation.

**Table 2:** Univariate and multivariate analysis of prognostic factors of OS.

**Factors**	**Univariate analysis**			**Multivariate analysis**	
	**HR (95 % CI)**	***P* value**		**HR (95 % CI)**	***P* value**
UBE2M (high vs. low)	1.871(1.020-3.433)	0.043		2.276(1.218-4.254)	0.010
TNM (III–IV vs. I–II)	4.461(2.347-8.478)	<0.001		2.966(1.409-6.243)	0.004
AFP (ng/mL) (>20 vs.≤20)	3.402(1.514-7.646)	0.003		3.309(1.313-7.034)	0.009
Tumor size (cm) (>5 vs.≤5)	2.286(1.260-4.147)	0.007		1.397(0.713-2.739)	0.330
Tumor differentiation (III–IV vs. I–II)	2.055(1.133-3.726)	0.018		1.129(0.694-2.429)	0.413
Tumor number (multiple vs. solitary)	1.194(0.645-2.211)	0.572		-	n.a.
Gender (male vs. female)	0.697(0.358-1.357)	0.288		-	n.a.
Age, years (≥ 55 vs.< 55)	0.828(0.451-1.519)	0.542		-	n.a.
HBsAg (positive vs. negative)	1.023(0.492-2.130)	0.951		-	n.a.
Cirrhosis (positive vs. negative)	1.029(0.611-2.394)	0.585		-	n.a.

n.a., not applicable.

**Table 3:** Univariate and multivariate analysis of prognostic factors of DFS.

**Factors**	**Univariate analysis**			**Multivariate analysis**	
	**HR (95 % CI)**	***P* value**		**HR (95 % CI)**	***P* value**
UBE2M (high vs. low)	2.305(1.261-4.213)	0.007		2.292(1.240-4.234)	0.008
TNM (III–IV vs. I–II)	7.442(3.845-14.402)	<0.001		7.520(3.426-16.507)	<0.001
Tumor size (cm) (>5 vs.≤5)	2.174(1.191-3.965)	0.011		0.920(0.437-1.936)	0.826
AFP (ng/mL) (>20 vs.≤20)	1.460(0.761-2.803)	0.255		-	n.a.
Tumor differentiation (III–IV vs. I–II)	1.018(0.552-1.878)	0.953		-	n.a.
Tumor number (multiple vs. solitary)	1.024(0.551-1.901)	0.941		-	n.a.
Gender (male vs. female)	1.332(0.618-2.871)	0.465		-	n.a.
Age, years (≥ 55 vs.< 55)	0.693(0.370-1.298)	0.252		-	n.a.
HBsAg (positive vs. negative)	1.227(0.569-2.645)	0.602		-	n.a.
Cirrhosis (positive vs. negative)	0.883(0.460-1.693)	0.708		-	n.a.

n.a., not applicable.

### UBE2M promotes cell proliferation and facilitates G1/S transition *in vitro*

**Figure 2:**
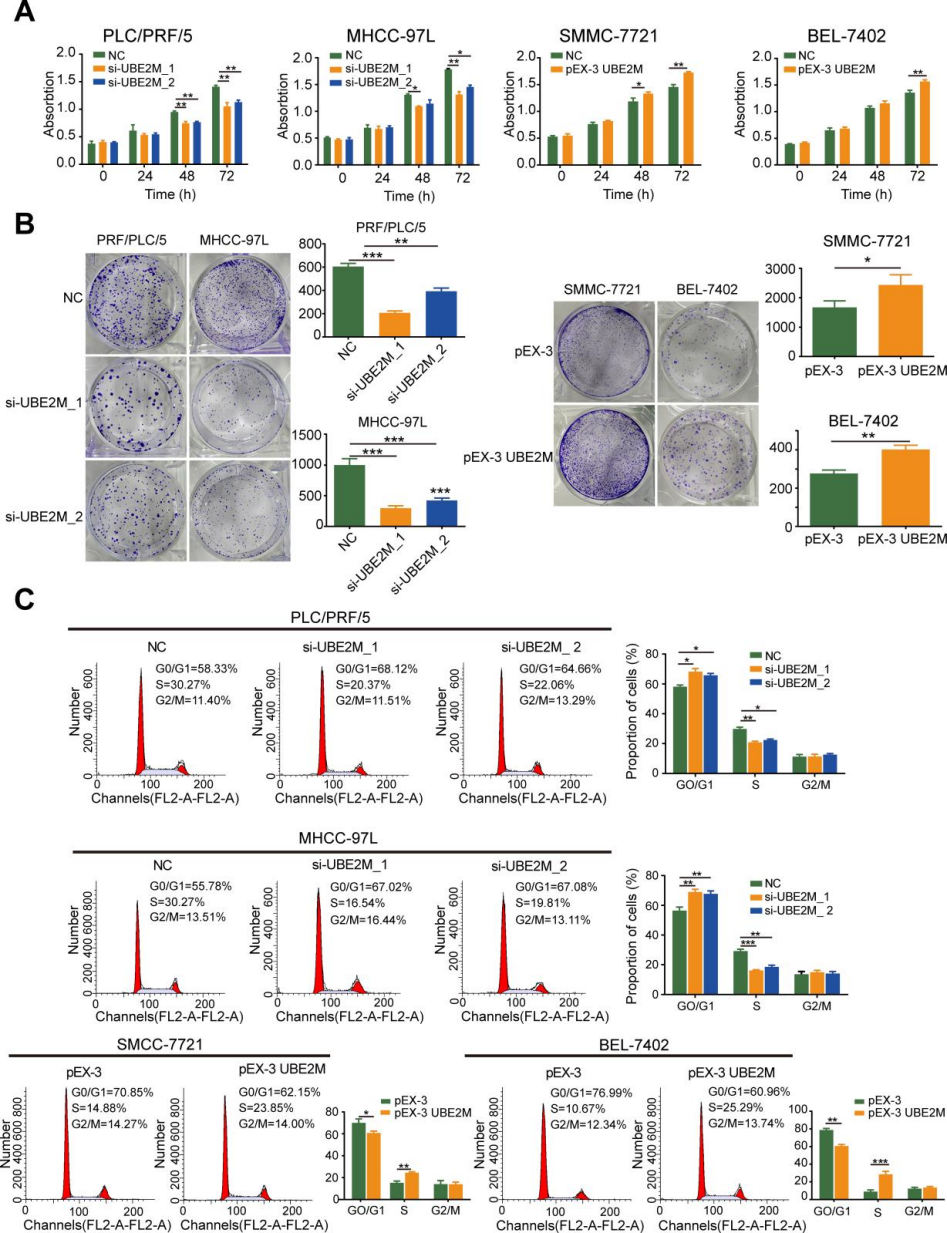
**UBE2M promotes cell proliferation *in vitro*.** (**A**) UBE2M knockdown significantly inhibited growth rate in UBE2M-silencing PLC/PRF/5 and MHCC-97L cells, while UBE2M overexpression significantly promoted growth rate in UBE2M-overexpressing SMCC-7721 and BEL-7402 cells, as revealed by CCK-8 assay. (**B**) The mean colony number was drastically reduced in UBE2M-silencing cells and was significantly increased in UBE2M-overexpressing cells, as determined by colony formation assay. (**C**) UBE2M knockdown significantly inhibited G1/S transition, while UBE2M overexpression remarkably induced G1/S transition, as measured by flow cytometry. Data were presented as mean ± SD (n=3). *P < 0.05; **P < 0.01; ***P < 0.001 (Student’s *t*-test).

To elucidate the biological function of UBE2M in the development and progression of HCC, we employed gain- and loss-of-function studies *in vitro*. Based on the UBE2M expression in HCC cell lines, we adopted PLC/PRF/5 and MHCC-97L cells with relative higher UBE2M expression for loss-of-function studies *in vitro,* while SMMC-7721 and BEL-7402 cells with relative lower UBE2M expression for gain-of-function assays *in vitro* (Supplementary Figure 2). By performing CCK-8 and colony formation tests, we observed that knockdown of UBE2M significantly suppressed proliferative capacity in PLC/PRF/5 and MHCC-97L cell lines compared with that in respective controls, while overexpression of UBE2M had the opposite effect in SMMC-7721 and BEL-7402 cells (Figure 2A and 2B).

Given that alterations in cell proliferation may be caused by changes in the cell cycle [19, 20], we tested the effect of UBE2M on cell cycle distributions by flow cytometry. The cell portion was substantially increased in the G0/G1 phase and decreased in the S phase after UBE2M suppression. In contrast, the cell portion was markedly reduced in the G0/G1 phase and raised in the S phase after UBE2M upregulation (Figure 2C). These data indicate that overexpressed UBE2M may promote cell proliferation by facilitating the G1/S transition in HCC.

### UBE2M promotes migration and invasion *in vitro*

**Figure 3:**
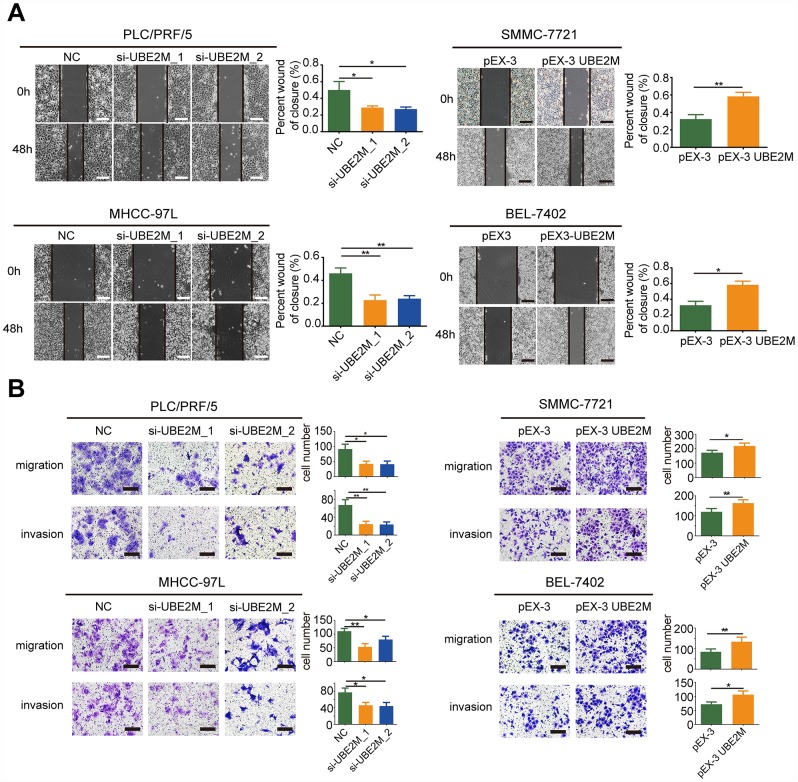
**UBE2M mediates cell migration and invasion *in vitro*.** (**A**) UBE2M knockdown remarkably suppressed cell migration in PLC/PRF/5 and MHCC-97L cells, while UBE2M overexpression significantly increased cell migration in SMMC-7721 and BEL-7402 cells, as demonstrated by wound-healing assay (scale bar, 50 μm). (**B**) Knockdown of UBE2M remarkably suppressed cell migration in PLC/PRF/5 and MHCC-97L cells, while overexpression of UBE2M significantly increased cell migration in SMMC-7721 and BEL-7402 cells, as determined by Transwell migration. Moreover, knockdown of UBE2M significantly reduced invasion capacity, while overexpression of UBE2M promoted invasion capacity, as revealed by Matrigel invasion assay (scale bar, 200 μm). Data was presented as mean ± SD (n = 3), *P < 0.05; **P < 0.01; ***P < 0.001 (Student’s *t*-test).

To further explore whether UBE2M could facilitate the process of metastasis, we also investigated the role of UBE2M in the regulation of cellular migration and invasion by performing the wound-healing assay and Transwell assay. The results revealed that knockdown of UBE2M markedly reduced cell migration, while overexpression of UBE2M considerably elevated cell migration (Figure 3A and 3B). Similarly, the Transwell assay demonstrated that knockdown of UBE2M significantly reduced invasion capacity, while overexpression of UBE2M actively promoted invasion capacity (Figure 3B). These data suggest that overexpressed UBE2M is sufficient to support HCC migration and invasion *in vitro*.

### UBE2M regulates cyclin D1 through stabilizing β-catenin

Based on the significant correlation between UBE2M expression and cell proliferation, we undertook to determine the mechanisms by which UBE2M induces cell proliferation. We assessed proteins responsible for G1/S transition, including cyclin E1, CDK2, and cyclin D1. We found that hypoxia or ectopic expression of UBE2M simply altered cyclin D1 expression, rather than cyclin E1 and CDK2 expression (Supplementary Figure 3). It is well known that cyclin D1 is a crucial downstream target of β-catenin [21, 22]. Thus, we hypothesized that UBE2M regulated cell cycle through β-catenin in HCC.

**Figure 4:**
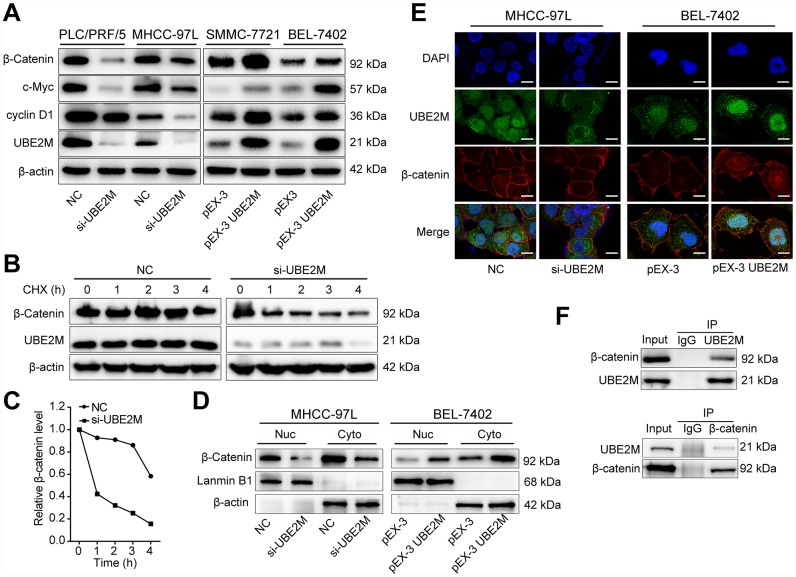
**UBE2M regulates the expression of cyclin D1 through stabilizing β-catenin.** (**A**) The protein expression level of β-catenin, cyclin D1, and c-Myc in UBE2M-silencing PLC/PRF/5 and MHCC-97L cells, as well as in UBE2M-overexpressing SMMC-7721 and BEL-7402 cells by Western blotting. (**B**) MHCC-97L cells transfected with NC siRNA and si-UBE2M for 48h, prior to addition of CHX (100 μg/mL). Cell lysates were prepared at the indicated times (0, 1, 2, 3, and 4 h) following addition of CHX, and analyzed by Western blotting. (**C**) Quantification of β-catenin protein levels in MHCC-97L cells at the indicated times as described in (**B**). (**D**) Nuclear and cytoplasmic fractions of MHCC-97L and BEL-7402 cells were isolated, and β-catenin expression was determined in the nuclear and cytoplasmic protein by Western blotting. (**E**) Nuclear and cytoplasmic expressions of UBE2M and β-catenin were analyzed by immunofluorescence staining in the MHCC-97L and BEL-7402 cells (Scale bar, 15 μm). (**F**) The physical interaction between UBE2M and β-catenin was examined by a co-IP assay in MHCC-97L cells. IgG was used as a negative control.

Firstly, qPCR results showed that there was no significant difference in the mRNA levels of β-catenin by either overexpressing or silencing UBE2M (Supplementary Figure 4). Next, β-catenin and its downstream targets, including cyclin D1 and c-Myc, were assessed by Western blotting. As predicted, the protein level of β-catenin, cyclin D1, and c-Myc was significantly decreased in UBE2M-suppressed PLC/PRF/5 and MHCC-97L cells, and was remarkably increased in UBE2M-overexpressed SMCC-7721 and BEL-7402 cells (Figure 4A). These results implied that UBE2M was involved in regulating β-catenin expression posttranscriptionally. Subsequently, downregulation of UBE2M exhibited a shorter half-life for β-catenin in the CHX chase assay, suggesting that UBE2M increases β-catenin protein stability in HCC cells (Figure 4B and 4C). Previous studies have demonstrated that when β-catenin is stabilized in cytoplasm, it would accumulate in the cytoplasm and is subsequently transported to the nucleus [23]. We also confirmed that upregulated UBE2M level remarkably promoted the accumulation of β-catenin and caused the translocation of β-catenin from the cytoplasm to the nucleus, while inhibition of UBE2M attenuated β-catenin level in both the cytoplasm and the nucleus (Figure 4D and 4E). Together, these results demonstrate that UBE2M increases the stability of β-catenin *in vitro*.

**Figure 5:**
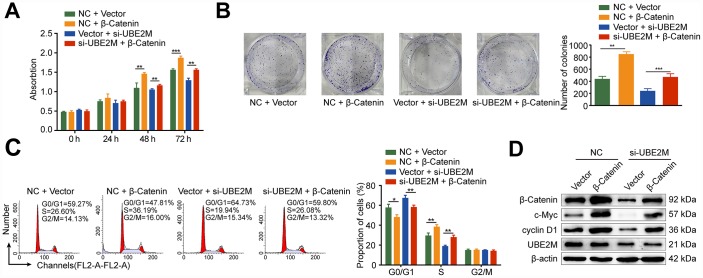
**Rescue from UBE2M-knockdown induced cell proliferation inhibition and cell cycle arrest by β-catenin overexpression.** MHCC-97L cells were co-transfected with the β-catenin overexpression plasmid or the empty expression plasmid in addition to the UBE2M vector or the empty expression vector. β-catenin overexpression enhanced cell proliferation and restored the suppressed effect caused by UBE2M knockdown, as revealed by CCK-8 assay (**A**) and colony formation assay (**B**). (**C**) β-catenin overexpression restored the inhibited G1/S transition caused by UBE2M knockdown determined by flow cytometry. (**D**) β-catenin, cyclin D1, c-Myc, and UBE2M expressions in MHCC-97L cells co-transfected with the β-catenin overexpression plasmid or the empty expression plasmid in addition to the UBE2M vector or the empty expression vector by Western blotting. Data was presented as mean ± SD (n = 3). *P < 0.05; **P < 0.01; *P* < 0.001 (student’s *t*-test).

Protein-protein interaction is a crucial mechanism to influence protein stability [24]. We, therefore, studied whether UBE2M co-operates directly with β-catenin via protein-protein communication. To verify the hypothesis, we performed immunofluorescence staining and co-IP assay to assess the interaction between the two proteins. Immunofluorescence staining showed that UBE2M colocalized with β-catenin in both the cytoplasm and the nuclear (Figure 4E), while co-IP assay showed that UBE2M could interact with β-catenin in MHCC-97L cells (Figure 4F). These results are in line with the previous study in HEK-293T cells [25]. To further confirm the critical role of β-catenin in UBE2M-induced proliferation, MHCC-97L cells were transfected by β-catenin cDNA plasmid or UBE2M RNAi alone or combined. The results of CCK-8 and colony formation assay demonstrated that co-transfection of β-catenin overexpression and UBE2M knockdown could partially restore the suppressed effect caused by UBE2M knockdown (Figure 5A and 5B). Consistently, the percentage of S phase was increased after MHCC-97L cells co-transfected with β-catenin overexpression plus UBE2M knockdown compared with cells treated with UBE2M knockdown (Figure 5C). Moreover, the result of Western blotting confirmed that overexpression of β-catenin could restore the reduction caused by UBE2M knockdown, which was further validated by the expression of downstream targets, including cyclin D1 and c-Myc (Figure 5D). Together, these findings suggest that UBE2M promotes cell proliferation through stabilizing β-catenin.

### UBE2M mediates tumor formation of HCC cells in a mouse xenograft model

**Figure 6:**
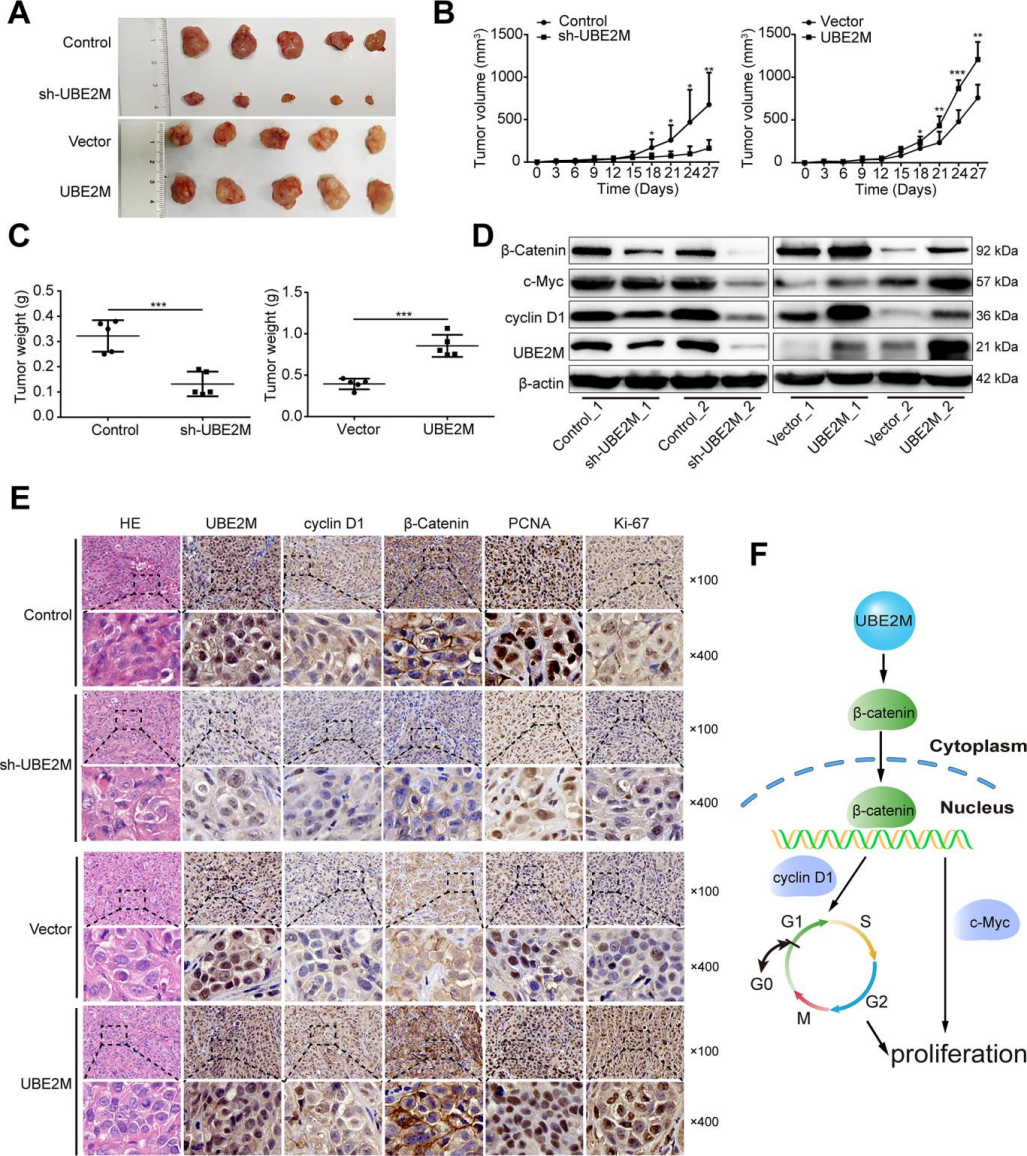
**UBE2M mediates tumor growth in a xenograft HCC model.** (**A**) Images of the tumors that develop in nude mice treated with UBE2M-knockdown MHCC-97L cells or control cells (upper panel), as well as UBE2M-overexpression BEL-7402 cells or control cells (lower panel). (**B**) Tumor volume was measured at the indicated time points, and the tumor growth curve was shown using a time-course line plot in the indicated group (n=5 per group). (**C**) Tumor weight was recorded on the 27^th^ day after the mice were sacrificed in the indicated group (n=5 per group). (**D**) The expression of UBE2M, cyclin D1, c-Myc, and β-catenin in the tumors of indicated group by Western blotting. (**E**) Representative Hematoxylin and eosin (HE) and immunohistochemical staining of HCC tumors for UBE2M, cyclin D1, β-catenin, anti-PCNA and Ki67 expression. (×100: Scale bar, 50 μm. ×400: Scale bar, 12.5 μm). (**F**) Schematic of working model. Upregulated UBE2M translocates accumulated β-catenin from the cytoplasm to the nucleus, thus activating downstream β-catenin/cyclin D1 signaling to promote cell cycle and proliferation. Data were expressed as mean ± SD (n = 5). *P < 0.05; **P < 0.01; ***P < 0.001 (student’s *t*-test).

To confirm the phenotype of UBE2M from *in vitro* studies, we further examined the effect of UBE2M on tumorigenesis using xenografts in nude mice with UBE2M-silencing MHCC-97L cells or UBE2M-overexpressing BEL-7402 cells. Mice with UBE2M-silencing cells significantly suppressed tumor growth and weight compared to vector-transduced mice (Figure 6A). Mean tumor volume was strikingly decreased from 675.54 ± 376.22 mm^3^ to 162.56 ± 97.46 mm^3^ (Figure 6B; *P* < 0.001); while mean tumor weight declined from 0.332 ± 0.06 g to 0.132 ± 0.04 g (Figure 6C; *P* < 0.0001). In contrast, mice with UBE2M-overexpressing cells remarkably upregulated tumor growth compared to vector-transduced mice (Figure 6A). Mean tumor volume was significantly increased from 759.05 ± 138.92 mm^3^ to 1207.05 ± 184.23 mm^3^ (Figure 6B; *P* < 0.01), while mean tumor weight increased from 0.394 ± 0.06 g to 0.855 ± 0.12 g (Figure 6C, *P* < 0.001). Moreover, the expression levels of UBE2M and its downstream targets (including cyclin D1, c-Myc, and β-catenin) in UBE2M-silencing mice was significantly reduced compared with that in vector-transduced mice, while their expression levels were significantly upregulated in UBE2M-overexpressing mice compared with that in vector-transduced mice (Figure 6D). Also, IHC staining illustrated that UBE2M-silencing mice showed limited staining for UBE2M, cyclin D1, PCNA, Ki67, and β-catenin compared with mice in control group, while UBE2M-overexpressing mice showed strong staining for these proteins relative to vector-transduced mice (Figure 6E and Supplementary Figure 5). Collectively, these results suggest that UBE2M promotes cell proliferation by facilitating cell-cycle progression *in vivo*.

## DISCUSSION

HCC is the most common liver malignancies, and understanding the molecular mechanisms implicated in its development is highly demanded. Here, we identify that UBE2M is upregulated in HCC tissues and cell lines, and shows potential predictive value for OS and DFS. Furthermore, overexpressed UBE2M promotes, while silencing UBE2M inhibits cell proliferation. Finally, we find that UBE2M is a crucial player in the G1/S transition of the cell cycle by stabilizing β-catenin and upregulating its downstream targets cyclin D1 expression *ex vivo* and *in vivo* (Figure 6F).

UBE2M, as an oncogene, has been confirmed to be overexpressed in several tumors, including osteosarcoma, urothelial carcinoma, cholangiocarcinoma, lung cancer, and HCC [16–18, 26, 27]. Consistent with previous studies, our results show that UBE2M is remarkably elevated in tumor tissues, which is in concordance with TCGA dataset. Although UBE2M overexpression has been identified, few studies have been conducted on its clinical significance in cancers. In the present study, we reveal that UBE2M overexpression is positively correlated with cirrhosis, tumor size, tumor number, and poor prognosis. Moreover, we find for the first time that elevated UBE2M expression could serve as a novel risk factor for poor prognosis of HCC.

Considering the remarkable correlation between UBE2M upregulation and advanced clinicopathological features of HCC, we further investigate the biological function of UBE2M *in vitro* and *in vivo*. Previous studies revealed that UBE2M promotes cell survival via the DNA damage repair process [28–30]. For example, Cukras et al. identified that UBE2M activates multiple Cullin ligases to promote cell cycle progression that is required for DNA damage response and maintaining genome integrity [30]. However, the phenotype and role of UBE2M in HCC remain mostly unknown. In the current study, we demonstrate that UBE2M promotes cell proliferation. This phenotype may be due to the fact that silencing UBE2M inhibits HCC proliferation and suppresses G1 to S phase transition, while overexpressing UBE2M promotes proliferation and activates G1 to S phase transition.

Moreover, we observe that UBE2M could regulate the expression of cyclin D1, a critical regulator of G1/S transition [31–33], which suggests a causative mechanism for UBE2M in HCC proliferation. Meanwhile, we observe that silencing UBE2M promotes cell apoptosis, while overexpressing UBE2M inhibits apoptosis (Supplementary Figure 6). The fact suggests that UBE2M may promote HCC proliferation by multiple cellular processes. In the present study, we focus on the mechanism of UBE2M regulating HCC proliferation through the cell cycle.

The Wnt/β-catenin pathway plays an essential role in cancer pathogenesis in which activation associated with tumorigenesis of over 50% HCC patients [34, 35]. Once the pathway is activated, β-catenin degradation is inhibited, leading to its accumulation in the cytoplasm and then nuclear translocation. Subsequently, the β-catenin in the nucleus promotes the transcription of cyclin D1, accelerating the G1/S transition of the cell cycle [8, 9]. In the study, we confirm this observation in HCC cells. In the rescue assay, β-catenin overexpression partially restores the suppressed effect caused by UBE2M knockdown, which further reinforces the crucial role of β-catenin in UBE2M-induced proliferation.

Protein-protein interactions serve a critical regulatory role in influencing protein stability. Previous studies have identified that the degradation process of β-catenin may be blocked by interacting with other proteins, like Mucin 16, Smad7, and BCL-9 [36–38]. In the present study, we examine the colocalization and interaction between UBE2M and β-catenin in HCC cells, which is partially consistent with a previous study performed in HEK-293T cells [25]. It should be noted that the detail mechanisms of their interactions remain to be further experimentally addressed. Irrespective of the unresolved questions, our studies provide the first evidence that UBE2M promotes cell proliferation via the UBE2M/β-catenin/cyclin D1 pathway in HCC. Besides revealing the mechanisms whereby UBE2M mediates clinical characteristics in HCC patients, our findings may also be helpful for the development of improved protocols that may benefit HCC patients with high UBE2M expression.

In summary, our study demonstrates a potentially significant role of UBE2M in promoting the growth of HCC and suggests its use as a possible therapeutic strategy for this malignancy.

## MATERIALS AND METHODS

### Clinical specimens and cell lines

Primary HCC and adjacent nontumoral tissues were obtained from 90 patients who underwent curative surgery for HCC between January 2015 and December 2015 at Zhongshan Hospital, Fudan University (Shanghai, China). Informed consent was obtained from each patient, and all procedures were approved by the Institute Ethics Committee.

PLC/PRF/5, BEL-7402, SMCC-7721, and L02 cell lines were obtained from the Cell Bank of the Chinese Academy of Science (Shanghai, China). MHCC-97L has been established in our institution [39]. These cells were cultured in Dulbecco’s modified Eagle’s medium (DMEM; Gibco, Carlsbad, CA, USA) containing 10% fetal bovine serum (FBS; Gibco, Carlsbad, CA, USA) and 1% penicillin/streptomycin (Corning, Lowell, MA, USA) at 37 °C with 5% CO_2_ as described previously [40].

### Cell transfection

Specific small interfering RNAs (siRNA) of UBE2M (RNAi#1: CAGAGGTCCTGCAGAACAA; RNAi#2: GGATCCAGAAGGACATAAA) were obtained from GenePharma Co. Ltd. (Shanghai, China). As a control, scrambled versions of these sequences were used. For the overexpression of UBE2M and β-catenin, full-length human UBE2M and β-catenin were cloned into a pEX-3 vector (GenePharma, Shanghai, China), respectively. Cellular transfection was performed by using Lipofectamine 3000 (Invitrogen, Carlsbad, CA, USA) according to the manufacturer's instruction. The transfected cells were treated with 100 μg/mL cycloheximide (CHX) (Sigma, Japan) for indicated times to measure β-catenin stability. Subsequently, these cells were collected for Western blotting analysis.

The RNAi and full-length sequences of human UBE2M were used for constructing lentiviral particles (GenePharma, Shanghai, China) to obtain UBE2M stably expressed cells. After 48 h of infection with lentivirus, the cells were transferred to medium supplemented with 4 μg/mL puromycin and were cultured for seven days. The efficiency of these lentivirus particles was validated, and the one with higher efficiency was adopted for subsequent study.

### Cell proliferation assay

Cell viability was determined using the Cell Counting Kit-8 (CCK-8) kit (Beyotime, Shanghai, China) according to the protocol. Briefly, 3×10^3^ cells were seeded in each well of 96-well plates and were cultured overnight. According to the instructions, CCK-8 reagent was added at indicated time points and incubated at 37°C for one h. The absorbance was measured at 490 nm using a microplate reader (MultiscanTMGO, Thermo, USA).

### Colony formation assay

Approximately 1,000 cells were seeded into a 6-well plate and cultured in medium containing 10% FBS for 14 days at 37°C. After removing the medium, colonies were fixed in methanol and stained with crystal violet (Beyotime, Shanghai, China) for visualization and counting.

### Cell cycle and apoptosis assays

For the cell cycle analysis, the cells were collected and fixed with ice-cold 70% ethanol overnight at 4 °C. After fixation, the cells were washed with phosphate-buffered saline (PBS) and subsequently incubated with propidium iodide (BD Bioscience, San Jose, CA, USA) for 30 min at room temperature. For cell apoptosis, it was analyzed using an Annexin V-FITC Apoptosis Detection Kit (BD Biosciences) according to the instructions of the manufacturer. Briefly, after washed twice with PBS, the cells were re-suspended in 100 μL binding buffer. Subsequently, the cells were incubated with 5 μL Annexin V-FITC and 5 μL propidium iodide for 20 min at room temperature. Flow cytometry was carried out by a FACS Calibur flow cytometer (BD Biosciences). Data acquisition and analysis were performed with CellQuest (BD Biosciences).

### Wound healing assay

For wound healing, cells were seeded into a 6-well plate. Cells were grown to confluency and were scratched with a 200 μL pipette tip. Subsequently, cells were cultured in DMEM medium without FBS. The speed of wound closure was photographed by microscope (Olympus, Tokyo, Japan) at indicated time points, and the migration ability was assessed by measuring the distance between the wound edges.

### Transwell assay

For the migration assay, cells in serum-free medium were seeded into the upper chamber of the Transwell, while for the invasion assays, the cells were plated on insert coated with Matrigel (Sigma Aldrich, USA). The bottom chamber was added with a medium with 10% FBS. After incubation at 37°C for 24 h (Migration) or 48 h (Invasion), the cells that had migrated to the underside of the membrane were fixed with methanol, stained with crystal violet and enumerated under an inverted microscope.

### RNA extraction and quantitative real-time PCR (qPCR)

The Trizol reagent (Invitrogen, USA) was used to extract total RNA and PrimeScriptTM RT Master Mix (TaKaRa, Dalian, China) were used for the reverse-transcription reactions according to the manufacturer's protocol. QRT-PCR was performed on an Applied Biosystems 7500 Real-time PCR system (Applied Biosystems, USA) with SYBR Premix Ex Taq II Kit (TaKaRa, Dalian, China). GAPDH was used as an internal control. The sequences of the primers used were as follows: UBE2M-forward primer: 5'-ATGAGGGCTTCTACAAGAGTGG-3'; UBE2M-reverse primer: 5'-ATTGTCTCACACTTCACCTTGG-3'; β-catenin-forward primer: 5'-GCGCCATTTTAAGCCTCTCG-3'; β-catenin-reverse primer: 5'-GGCCATGTCCAACTCCATCA-3'; GAPDH-forward primer : 5'-ACCACAGTCCATGCCATCAC-3'; GAPDH-reverse primer: 5'-CCACCACCCTGTTGCTG-3'.

### Immunohistochemical (IHC) analysis

The slides with paraffin-embedded tissues were dried at 60°C for 30 min and washed with xylene to deparaffinization. Next, tissues were rehydrated with graded alcohols. After the endogenous peroxidase activity was blocked with 3% hydrogen peroxide for bright background staining, the slides were incubated with anti-UBE2M antibody overnight at 4°C. Subsequently, the secondary antibody was used. Finally, the slides were stained with diaminobenzidine counterstained with 20% hematoxylin.

All specimens were histologically examined by two independent pathologists, as we described previously [41]. Briefly, positive immunoreactivity for UBE2M was presented as brown staining and localized in both nucleus and cytoplasm. For the assessment of UBE2M, a semiquantitative histochemistry score (H-score) system was adopted and based on the percentage of positively staining cells (0, 0% positive cells; 1, 1–25% positive cells; 2, 26–50% positive cells; 3, 51–75% positive cells; 4, 76–100% positive cells) and the staining intensity (0, negative; 1, weak; 2, intermediate; 3, strong). The final expression score was generated by multiplying the scores of the percentage of positive cells and staining intensity, with a full range from 0 to 12. Samples with a sum score > 6 were considered as high UBE2M expression while those with a sum score ≤ 6 were defined as low UBE2M expression. Interobserver disagreement cases were reviewed once more to reach a conclusive judgment.

### Co-Immunoprecipitation (co-IP) and Western blotting

Co-IP was carried out using protein G-agarose (Millipore, Billerica, MA, USA). Western blotting was performed as previously described [42]. Briefly, cultured cell or frozen tissues lysed in radio-immunoprecipitation assay (RIPA) buffer supplemented with 1% protease inhibitor. The following antibodies were from Cell Signaling Technology (Danvers, MA, USA) unless otherwise noted: anti-β-actin, anti-β-catenin, anti-cyclin D1, anti-CDK2, anti-cyclin E1, anti-c-Myc, anti-UBE2M (Abcam, Cambridge, MA, USA), and anti-lanminB1 (Proteintech, Rosemont, IL, USA).

### Immunofluorescence staining

Cells seeded on coverslips in 12-well plates were fixed with 4% paraformaldehyde, permeabilized with 0.1% Triton X-100, and blocked with 5% bovine serum albumin. After that, the cells were incubated with anti-UBE2M and anti-β-catenin at 4 °C for overnight. Subsequently, Alexa Fluor 594- and 488-conjugated secondary antibodies (Invitrogen, Carlsbad, CA) were incubated with the cells at 37 °C for one h. The cells were stained with 4',6-diamidino-2-phenylindole (DAPI), and photographed using a Leica SP8 confocal microscope (Leica, Germany).

### Animal experiments

Animal experiments were approved by the Animal Ethics Committee of Zhongshan Hospital, Fudan University (Shanghai, China). Twenty 5-week-old BALB/C-nu/nu nude mice were randomly allocated into four groups. Approximately 5×10^6^ cells (MHCC-97L control or MHCC-97L sh-UBE2M) or 3×10^6^ cells (BEL-7402 vector or BEL-7402 UBE2M) suspended in 200 μL serum-free medium were subcutaneous injected directly into the right dorsal flank per mouse. The developing tumors were observed over the next four weeks and measured with calipers every three days. The tumor volumes were calculated using the formula: 0.5 × (length × width^2^). After mice were euthanized, tumors were removed and weighed at the end of follow-up.

### TCGA data acquisition

UBE2M expression and appropriate survival time of HCC patients in TCGA database were acquired from the visualized websites. UBE2M expression in tumor and non-tumor tissues was obtained from UALCAN (http://ualcan.path.uab.edu/index.html). Overall survival and disease-free survival time in high and low UBE2M group were acquired from GEPIA (http://gepia.cancer-pku.cn/index.html).

### Statistical analysis

Statistical analyses were conducted using GraphPad Prism 6.0 (GraphPad Prism, La Jolla, CA, USA) and SPSS 22.0 software (SPSS Inc., Chicago, USA). Kaplan-Meier method and the log-rank test were used for generating survival curves. The Student's *t*-test was used for statistical comparisons between two experimental conditions, and the chi-square test was applied to assess correlations between UBE2M expression and clinicopathological features of HCC patients. *P* <0.05 was considered statistically significant.

## Supplementary Material

Supplementary Figures
