# Supplementary material for: UBE2M promotes cell proliferation via the β-catenin/cyclin D1 signaling in hepatocellular carcinoma
Source: Aging (Albany NY). 2020 Feb 3;12(3):2373–92. doi: 10.18632/aging.102749 (PMC7041726; doi:10.18632/aging.102749)
Supplement: Supplementary Figures [file aging-12-102749-s001..pdf]

SUPPLEMENTARY FIGURES

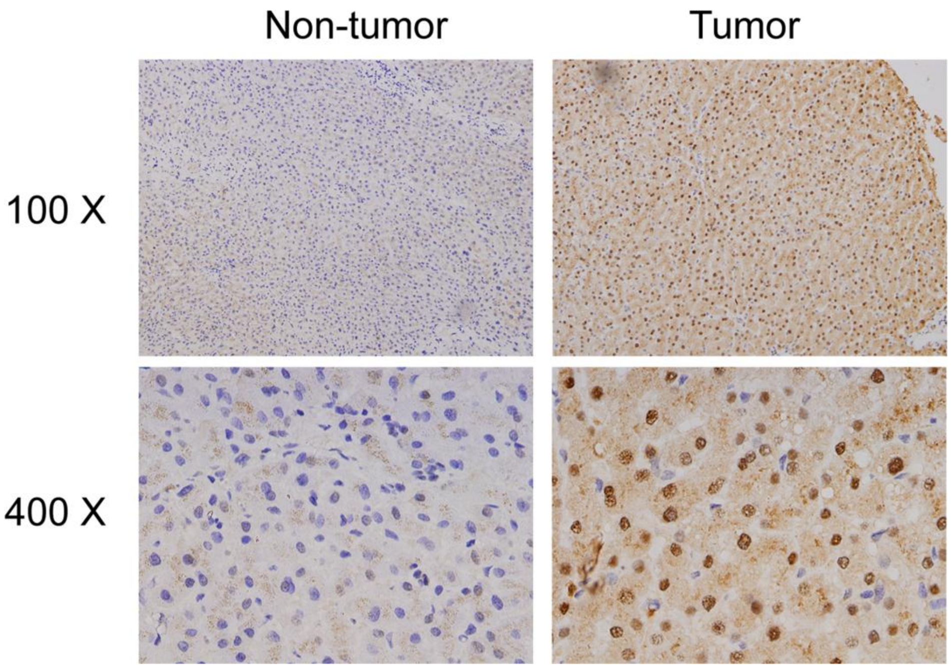

Supplementary Figure 1. Representative images of UBE2M expression in HCC and non-tumor tissues (Scale bar, 100 X in upper panel and 400 X in lower panel).

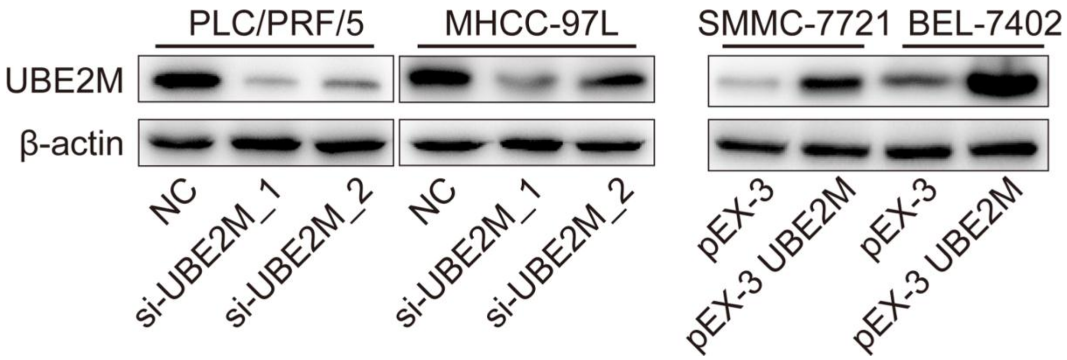

Supplementary Figure 2. Transfection efficiency of siRNAs and overexpression plasmids in the indicated cells was verified by Western Blotting.

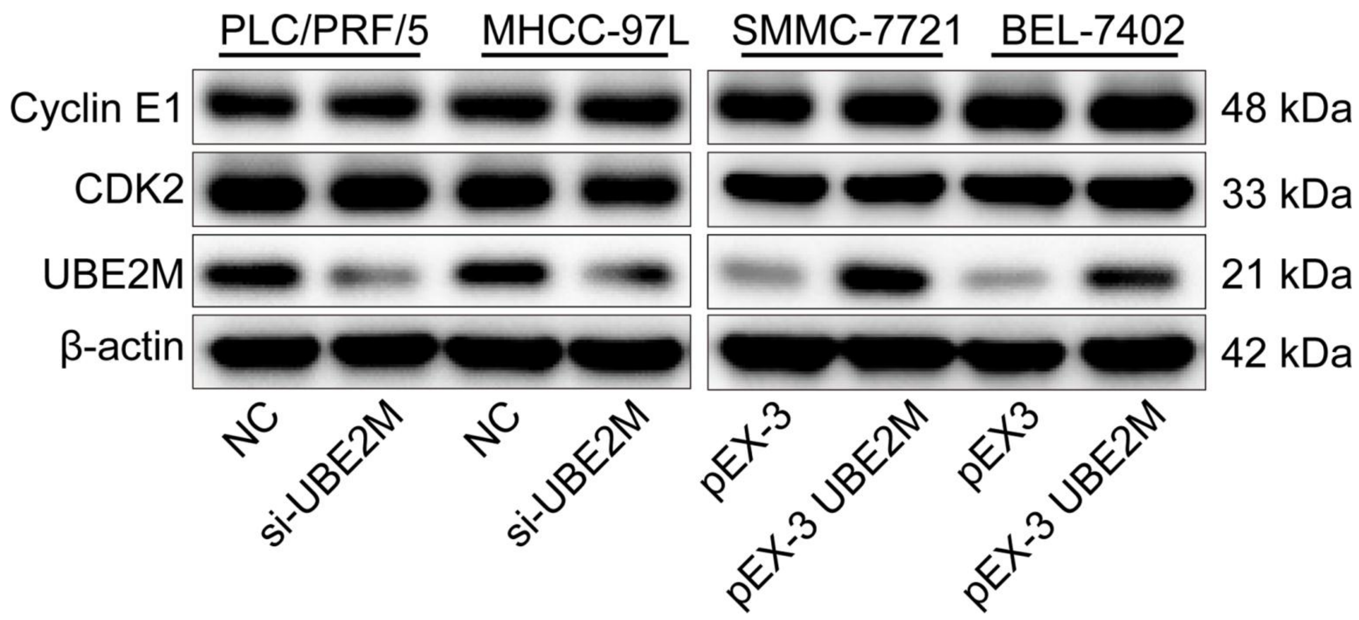

Supplementary Figure 3. The expression levels of cyclin E1 and CDK2 proteins was detected by Western Blotting when UBE2M was knocked down or overexpressed.

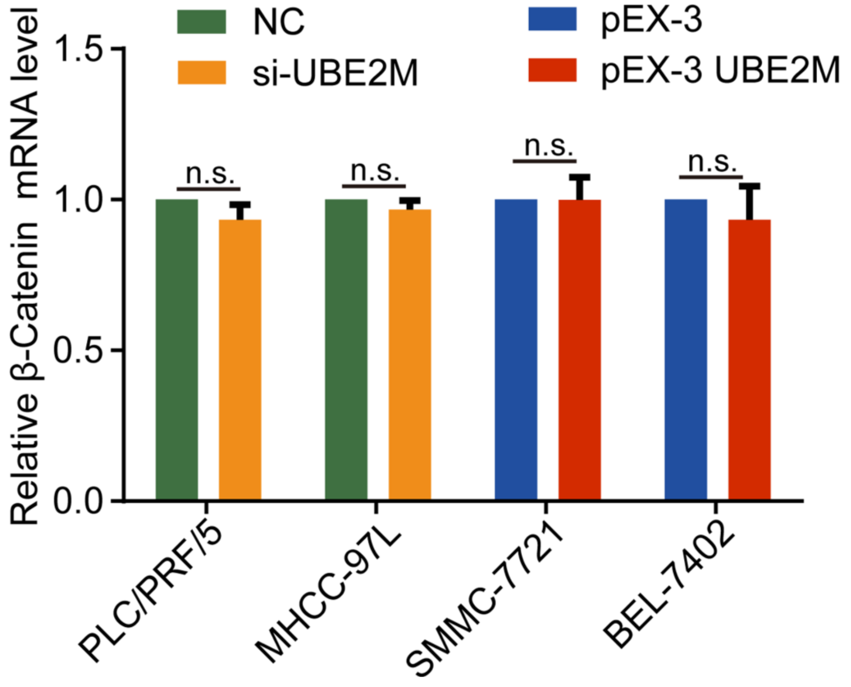

Supplementary Figure 4. The mRNA level of β-catenin was assessed by qPCR in UBE2M-silenced or -overexpressed cells, as well as corresponding control cells. Data was presented as mean ± SD (n = 3). n.s., not significant. (Student's *t*-test).

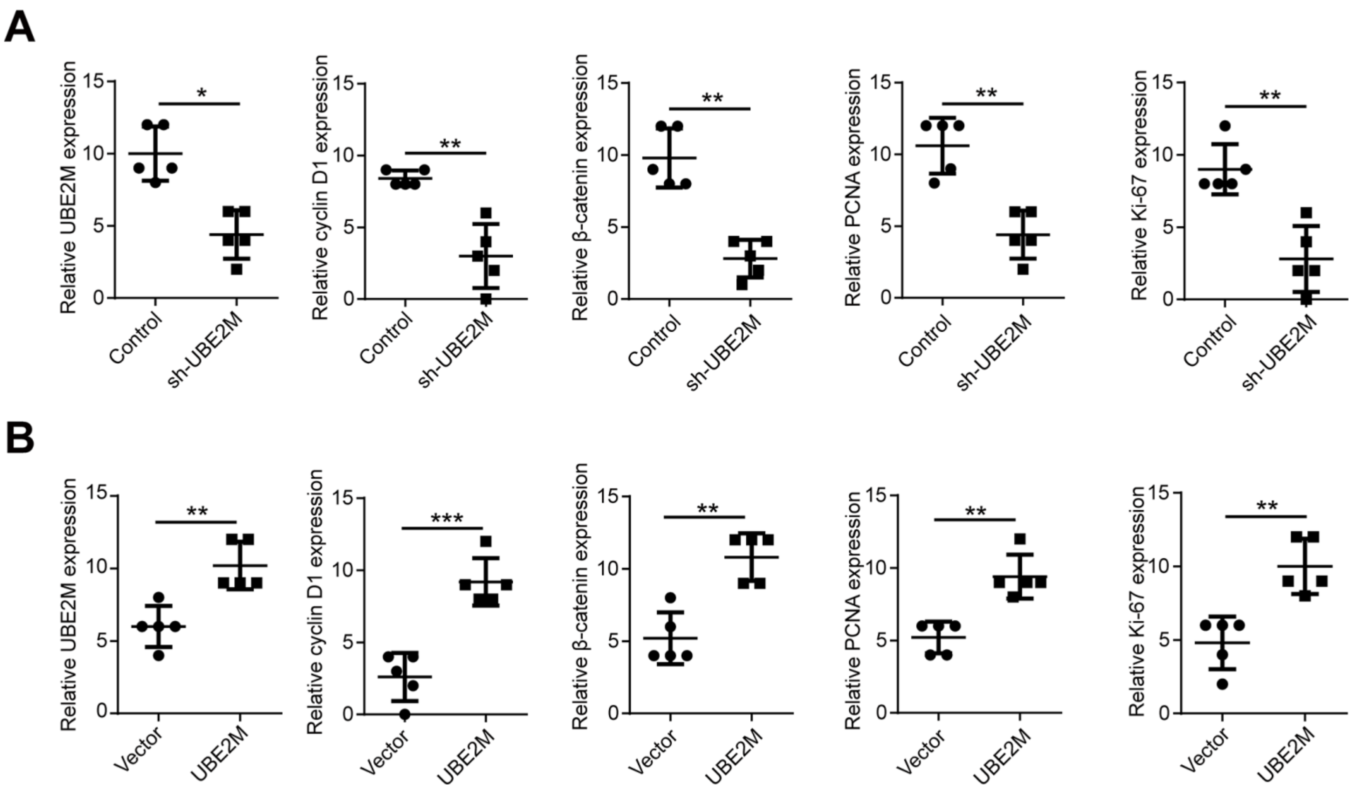

Supplementary Figure 5. Quantification of the IHC staining for indicated proteins by histochemistry score. (**A**) Relative expression of UBE2M, cyclin D1, β-catenin PCNA and Ki67 in the control and sh-UBE2M groups. (**B**) Relative expression of UBE2M, cyclin D1, β-catenin PCNA and Ki67 in the vector and UBE2M groups. Data were presented as mean ± SD (n=5). *$P < 0.05$; **$P < 0.01$; ***$P < 0.001$ (Student's *t*-test).

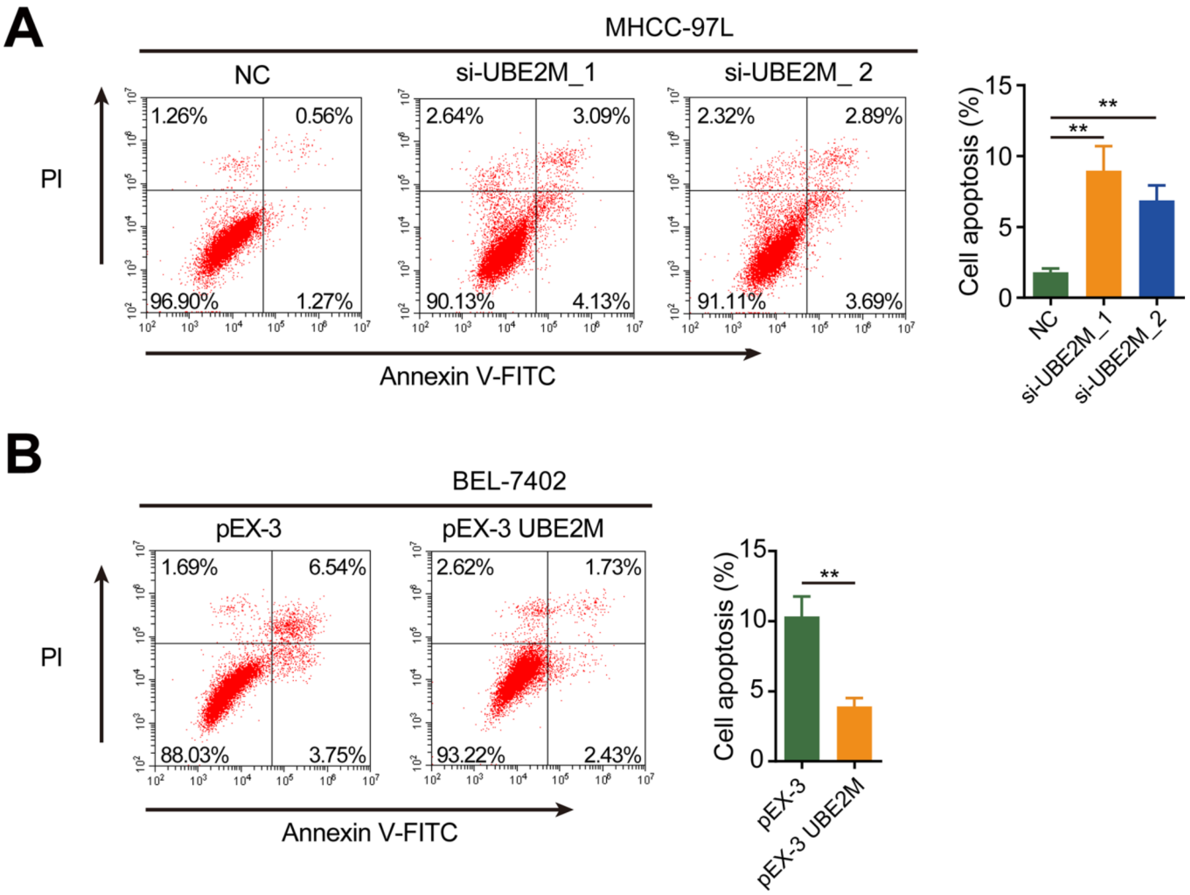

Supplementary Figure 6. UBE2M knockdown significantly promoted apoptosis (**A**), while UBE2M overexpression remarkably inhibited apoptosis (**B**), as measured by flow cytometry. Data were presented as mean ± SD (n=3). ***P* < 0.01 (Student's *t*-test).
